# Analyses of receptor binding specificity of highly pathogenic avian influenza A (H5N1) viruses isolated from felines in South Korea, 2023

**DOI:** 10.1080/21505594.2026.2636350

**Published:** 2026-02-23

**Authors:** Dong-Hyun Son, Anand Balupuri, Jeong-Hyun Nam, Il-Hwan Kim, Yong Jun Choi, Bo Min An, Jeong-Min Kim, Eun-Jin Kim, Nam Sook Kang

**Affiliations:** a Graduate School of New Drug Discovery and Development, Chungnam National University, Daejeon, Republic of Korea; b Division of Emerging Infectious Diseases, Department of Laboratory Diagnosis and Analysis, Korea Disease Control and Prevention Agency, Cheongju, Republic of Korea

**Keywords:** H5N1 influenza virus, strains, outbreak, receptor binding specificity, transmission potential

## Abstract

Influenza viruses infect host cells by binding to specific sialic acid receptors present on the surface of target cells, and this receptor binding exhibits specificity depending on cell type and host species. Avian influenza A (H5N1) viruses typically bind preferentially to α2,3-linked sialic acid receptors, although some strains have been reported to acquire binding affinity for the human-type α2,6-linked sialic acid receptors, highlighting the need for ongoing receptor binding analyses of highly pathogenic avian influenza (HPAI) viruses. Notably, in July 2023, two distinct cases of fatal cluster infections in felines caused by HPAI H5N1 viruses were reported for the first time in South Korea (Gwanak and Yongsan). Characterization of the isolated strains revealed high pathogenicity and efficient contact transmission in mammals. In this study, we investigated the receptor binding specificity of the H5N1 viruses associated with these feline outbreaks to assess their potential threat to human health. Our findings demonstrated that both felines-derived and avian-derived H5N1 isolates retained strong binding affinity to avian-type α2,3-linked sialic acid receptors, while showing no detectable binding to human-type α2,6-linked sialic acid receptors. These results provide experimental evidence that the feline H5N1 isolates retain avian-type receptor specificity, indicating a low potential for efficient human-to-human transmission.

## Introduction

Highly pathogenic avian influenza (HPAI) is an infectious disease primarily affecting avian species, often causing high mortality. Certain subtypes, however, can cross species barriers and infect mammals, including humans, raising serious public health concerns. Since the first human infection in Hong Kong (1997), H5N1 viruses have spread extensively across Asia, Europe, Africa, and North America, posing repeated zoonotic threats [1–4].

Among currently circulating HPAI viruses, the H5N1 subtype belonging to clade 2.3.4.4b has become predominant globally, with confirmed cases of spillover into mammals in addition to wild birds [1,2]. The 2.3.4.4b H5N1 lineage was first identified in wild ducks in 2021 and has since been consistently detected in both wild birds and domestic poultry farms across the Republic of Korea [5]. More recently, fatal cluster infections of felines caused by H5N1 2.3.4.4b viruses were reported at two felines shelters in Seoul. Virological characterization of the isolates revealed high pathogenicity and efficient contact transmission in mammalian hosts, highlighting the need for further assessment of their zoonotic potential [6].

Influenza viruses infect through the binding of their envelope glycoprotein, hemagglutinin (HA), to terminal sialic acid (SA) residues on host cell surfaces. Avian influenza viruses preferentially bind to α2,3-linked SA receptors, whereas human influenza viruses bind primarily to α2,6-linked SA receptors [7,8]. This receptor binding preference serves as a major interspecies transmission barrier for influenza viruses. Although human-to-human transmission of 2.3.4.4b HPAI H5N1 remains limited, continued report of sporadic zoonotic infections highlights that receptor binding specificity remains a critical determinant of host adaptation and transmissibility [4,7–17]. Consequently, monitoring whether clade 2.3.4.4b H5N1 viruses isolated in South Korea in 2023 acquired human-type (α2,6-linked) SA receptor affinity is essential for real-time assessment of zoonotic risk.

While receptor binding properties of H5N1 viruses have been investigated extensively, most studies have not directly addressed recent mammalian spillover events in natural outbreak settings. In this study, we therefore adopted a comparative, host- and region-specific approach to evaluate whether the species jump from birds to felines, and subsequent transmission among felines, was accompanied by detectable changes in receptor binding specificity. Specifically, we compared feline-derived clade 2.3.4.4b H5N1 viruses isolated in South Korea in 2023 with a genetically related avian H5N1 strain isolated from ducks in the same region.

To achieve this, we analyzed the receptor binding specificity of HPAI H5N1 viruses associated with feline mortality clusters reported in South Korea using a combined experimental and computational approach. Receptor binding assays were conducted to assess interactions with avian-type (α2,3-linked) and human-type (α2,6-linked) SA receptors, with an H1N1 strain known to preferentially bind human-type (α2,6-linked) SA receptor included as a reference control [18]. To support and interpret these experimental observations, we performed computational modeling, molecular dynamics (MD) simulations, and Molecular mechanics/Poisson – Boltzmann surface area (MM/PBSA) analyses of HA-receptor complexes. Given the rapid evolution of influenza viruses, providing updated, region-specific data on feline H5N1 isolates from South Korea in 2023 fills an important gap in the global surveillance of clade 2.3.4.4b viruses currently driving unprecedented outbreaks worldwide.

## Material and methods

### Source and isolation of influenza virus strains

H5N1 influenza virus A/duck/Korea/H493/2022 (Duck/YC/2022, GISAID accession no. EPI_ISL_15647834) isolated from a farmed duck in October 2022, was obtained from the Animal and Plant Quarantine Agency. Additionally, two H5N1 influenza virus isolates from felines, obtained from outbreaks in Seoul, A/feline/Korea/M305-7/2023 (Feline/GA/2023, GISAID accession no. EPI_ISL_18819807) from an animal shelter in Gwanak District and A/feline/Korea/M302-6/2023 (Feline/YS/2023, GISAID accession no. EPI_ISL_18819809) from Yongsan District in July 2023 were also provided by the same agency (Table S1). As a control representing high affinity for human-type receptors, the 2009 pandemic H1N1 virus (A/California/04/2009, pdm09. GISAID accession no.EPI_ISL_29573) was used (Table S1).

### Receptor binding assay for viruses

To evaluate the receptor binding affinity of influenza viruses Feline/GA/2023, Feline/YS/2023 and Duck/YC/2022, a solid-phase direct-binding assay was performed using the following biotinylated sialylglycan receptors purchased from GlucoNZ (USA): α2,3-SLN (Neu5Acα2-3 Galβ-4GalNAcα-PAA-Biotin), α2,3-SL (Neu5Acα2-3 Galβ-4Galcα-PAA-Biotin), α2,6-SLN (Neu5Acα2-6 Galβ-4GalNAcα-PAA-Biotin) and α2,6-SL sialyl linkages (Neu5Acα2-6 Galβ-4Galcα-PAA-Biotin). Briefly, each virus sample (32 hemagglutinating units) was added to 96-well microtiter plates coated with 20 μg/ml Fetuin (Sigma, USA) and incubated overnight at 4°C. After removal of the virus inoculum, wells were blocked with PBS containing 5% BSA at room temperature for 1 hour. Plates were washed with ice-cold PBST, biotinylated glycans diluted to 5 μM in reagent containing 5 μM Oseltamivir acid (CAYMAN CHE, USA) were added and incubated at 4°C for 2 hr. Following another wash with ice-cold PBST, horseradish peroxidase (HRP)-conjugated streptavidin (1:1000 dilution in PBS) was added to each well and incubated at 4°C for 1 hr. After a final wash with ice-cold PBST, 0.1 ml of 3,3′,5,5′-tetramethylbenzidine (TMB) substrate was added to each well and incubated at room temperature for 10 min. The reaction was stopped by adding 0.05 ml of 50 mM hydrochloric acid (HCl). Optical density at 450 nm was measured using a Multiskan Sky multilabel plate reader (Thermo Scientific, USA). Human pandemic H1N1 (pdm09) virus (GISAID accession no.EPI_ISL_29573) was used as a positive control for α2,6-linked SA receptor binding.

### Statistical analysis of experimental data

Statistical analyses were performed using GraphPad PrismTM software (v9 or 10) (La Jolla, CA, USA).

### Structural modeling of viral strains

We modeled four distinct hemagglutinin (HA) proteins, three from different H5N1 strains (Feline/GA/2023, Feline/YS/2023 and Duck/YC/2022) and one derived from an H1N1 strain (pdm09) (Table S1). The H1N1 strain was included as a reference due to its established preference for human receptors, allowing for comparative analysis with the H5N1 strains. The HA protein sequences of all influenza viruses were obtained from the GISAID (supplementary data 2). Since no experimental structures were available for these H5N1 strains, their 3D structures were constructed by homology modeling. We used the crystal structure of H5N1 HA protein A/Vietnam/1196/2004 (PDB 3ZNM, resolution 2.4 Å) as the template which shares 92.0–92.2% sequence identity with the three H5N1 sequences [19]. The full-length HA sequences of the three H5N1 strains, except the signal peptide region (residue 1–16) and cleavage site (residue 338–346), were used as input for homology modeling. The 3D structure of the H1N1 HA protein (A/California/04/2009, pdm09) was modeled based on the crystal structure of H1N1 HA protein A/California/04/2009 (PDB 3UBQ, 2.0Å) that shares 99.5% sequence identity [20]. The homology models were built using the SWISS-MODEL web server [21].

To generate HA-glycan complexes, we used the structures of the avian receptor glycan analog α2,3-SLN and the human receptor glycan analog α2,6-SLN. The atomic coordinates for these glycans were extracted from existing HA – glycan co-crystal structures. Specifically, for H5N1 HA, the avian receptor was obtained from the H5N1 HA protein complex of A/turkey/Turkey/1/2005 (PDB 4BH1), while the human receptor was derived from its corresponding complex (PDB 4BH0) [22]. Similarly, for H1N1 HA, the avian receptor was retrieved from the H1N1 HA complex of the A/California/04/2009, pdm09 (PDB 3UBQ), while the human receptor was taken from its corresponding complex (PDB 3UBE) [20]. The extracted glycans were then placed onto the modeled HA structures by superimposing each target HA structure onto the corresponding glycan-bound template. All structural superimpositions and preparations were carried out using BIOVIA Discovery Studio V24 (BIOVIA, San Diego, CA, USA). To ensure computational efficiency, all MD simulations were performed using a monomeric form of the HA protein, although its native state is a homotrimer.

### Molecular dynamics simulations of viral complexes

MD simulations were performed using the GROMACS 5.1.4 software package [23]. The AMBER99SB-ILDN force field [24] was used for the protein, and the GLYCAM-web force field [25] was used for the glycan. Each system was placed in a cubic simulation box with a minimum distance of 15 Å from the box edges. The systems were solvated using the TIP3P water model and the net charge was neutralized by adding appropriate numbers of Na^+^ and Cl^−^ ions. To eliminate any steric clashes and optimize the initial structure, energy minimization was performed using the 50,000 steps of steepest descent algorithm. This was followed by a two-phase equilibration process. The first phase involved temperature equilibration under the isothermal – isochoric (NVT) ensemble for 100 ps. The second phase involved pressure equilibration under the isothermal – isobaric (NPT) ensemble for 200 ps. Temperature was maintained at 300 K using the V-rescale thermostat [26], while pressure was regulated at 1 atm using the Parrinello – Rahman barostat [27]. After equilibration, a production MD run was carried out for 200 ns under periodic boundary conditions and trajectory data were recorded every 10 ps. The leap-frog integrator [28] was used with a time step of 2 fs. All bonds involving hydrogen atoms were constrained using the LINCS algorithm [29]. Long-range electrostatic interactions were computed using the Particle Mesh Ewald method [30], while short-range interactions were truncated at a cutoff distance of 12 Å. Post-simulation analyses were conducted using standard GROMACS utilities [23]. Specifically, root-mean-square deviation (RMSD), root mean square fluctuation (RMSF) and angle θ calculations were carried out using the gmx rms, gmx rmsf and gmx angle tools, respectively.

### Binding free energy calculations via MM/PBSA

Binding free energy calculations were performed using the gmx_MMPBSA 1.5.2 package, which implements the MM/PBSA method [31]. The required input structures and trajectories were derived from MD simulations conducted using GROMACS. The dielectric interface was defined using the level-set function [32], while the nonpolar solvation free energy was estimated based on the solvent-accessible surface area (SASA) model [33]. The external and internal dielectric constants were set to 80 and 1, respectively.

### Intermolecular hydrogen bonding in viral complexes

H-bond interactions between the HA protein and the SLN glycan were examined throughout the MD simulation using the “Hydrogen Bonds” plugin available in Visual Molecular Dynamics (VMD) version 1.9.4 [34]. H-bonds were identified using the Luzar and Chandler criteria [35], which define an H-bond by the presence of suitable donor and acceptor atoms, with a distance cutoff of 3.5 Å and an angle cutoff of 30°. The occupancy of each identified H-bond was calculated, indicating the percentage of MD frames in which it appeared.

## Results

### Characterization of influenza virus isolates

Within the H5N1 HA protein, Feline/GA/2023, Feline/YS/2023 and Duck/YC/2022 share a common set of mutations including K36T, D45N, R53K, N72R, K82R, V86A, D94S, F95L, Q115L, S123P, S124N, A127T, S133A, K140A, S141P, N154D, S155D, T156A, R162I, Q169R, V174I, P181S, D183N, A185E, K189N, Q192K, R212K, K218Q, S223R, E227D, N236D, N240H, E268G, L269V, N273H, M282V, R310K, Q322L, R325K, K329del, D488Y, I513T, V523A and V533M. The primary difference among these strains lies at the position 83 (H5 numbering), Feline/GA/2023 carries A83N mutation, Feline/YS/2023 contains A83D mutation and Duck/YC/2022 retains the unmutated residue at this position.

### Analyses of receptor binding specificity by solid-phase direct binding assay

Assessing the human receptor binding affinity of HA is a critical parameter for evaluating the potential of influenza viruses to transmit efficiently among mammalian hosts. We evaluated the receptor binding preferences of the two 2023 feline isolates (Feline/GA/2023 and Feline/YS/2023) as well as the 2022 avian isolate (Duck/YC/2022).

**Figure 1.**
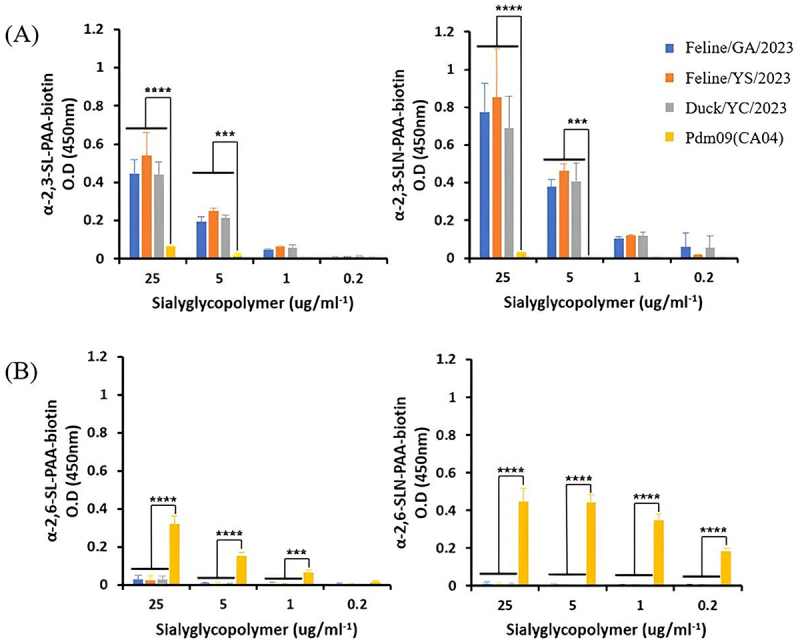
Receptor binding assay using sialyglycopolymers. The dose-dependent direct binding affinities of Feline/GA/2023, Feline/YS/2023 and Duck/YC/2022 viruses to (A) α2,3-SLN-PAA-biotin and α2,3-SL-PAA-biotin, (B) α2,6-SLN-PAA-biotin and α2,6-SL-PAA-biotin glycans. For comparison of receptor binding specificity, the pdm09 (A/California/04/2009) virus was used as a positive control for mammalian viruses. Statistical analyses were performed using the two-way ANOVA followed by Tukey’s multiple comparisons test with GraphPad Prism software (***p < 0.001, ****p < 0.0001).

In addition to α2,3-SLN and α2,6-SLN, receptor binding assays were also conducted using α2,3-SL (Neu5Acα2-3 Galβ1-4Glc) and α2,6-SL (Neu5Acα2-6 Galβ1-4Glc), yielding similar results. All three H5N1 viruses exhibited strong binding affinity to avian-type α2,3-linked sialic glycans, including α2,3-SLN and α2,3-SL, with binding intensities approximately 7–29 times higher than that of the pdm09 virus at 25 µg/mL glycan concentration. In contrast, these viruses showed little to no detectable binding to human-type α2,6-linked sialyl glycans, including α2,6-SLN and α2,6-SL, with binding signals approximately 10–61 times lower than pdm09 under the same conditions (Figure 1).

### Strain-specific structural stability patterns

We performed MD simulations for HA complexes with α2,3-SLN and α2,6-SLN glycans. To evaluate the stability of the simulated systems, we computed the RMSD of the protein backbone (Figure S1) and the receptor binding site (RBS) (Figure S2) with respect to their initial structures. The RMSD plots suggest that the simulated systems remained reasonably stable during the simulation, with notable fluctuations observed in the pdm09—α2,3-SLN and Duck/YC/2022—α2,6-SLN systems. Comparison of the average RMSD and standard deviation (SD) revealed that the pdm09 α2,6-SLN was more stable than α2,3-SLN complex. In contrast, for the other systems (Feline/GA/2023, Feline/YS/2023 and Duck/YC/2022), the α2,3-SLN complexes were more stable than their α2,6-SLN complexes (Table 1).

**Table 1.** Average RMSDs and standard deviations of the HA protein and its RBS, HA1 and HA2 subunits during MD simulations.

Virus – glycan complex	Protein (Å)	RBS (Å)	HA1 (Å)	HA2 (Å)
pdm09—α2,3-SLN	4.10 ± 1.34	1.15 ± 0.12	2.73 ± 0.59	4.31 ± 1.12
pdm09—α2,6-SLN	2.84 ± 0.45	1.04 ± 0.17	2.36 ± 0.31	3.59 ± 0.45
Feline/GA/2023—α2,3-SLN	2.78 ± 0.86	1.09 ± 0.17	2.40 ± 0.49	3.14 ± 0.41
Feline/GA/2023—α2,6-SLN	3.16 ± 0.66	1.85 ± 0.15	2.79 ± 0.47	3.46 ± 0.32
Feline/YS/2023—α2,3-SLN	4.36 ± 0.71	1.09 ± 0.09	3.39 ± 0.51	3.96 ± 0.35
Feline/YS/2023—α2,6-SLN	5.01 ± 1.05	1.31 ± 0.13	3.80 ± 0.71	3.96 ± 0.45
Duck/YC/2022—α2,3-SLN	2.89 ± 0.46	1.01 ± 0.13	2.31 ± 0.32	3.75 ± 0.54
Duck/YC/2022—α2,6-SLN	3.53 ± 0.99	1.02 ± 0.13	2.66 ± 0.49	3.54 ± 0.49

As our primary interest was the HA – glycan interface, we focused on a localized analysis of the receptor-binding site (RBS), defined as residues within 5 Å of the SLN ligand. In contrast to the whole protein structure, which showed some flexibility, the RBS remained highly stable across all systems, with average RMSD values consistently below 2 Å (Table 1 and Figure S2). This indicates that the RBS was well-equilibrated and suitable for detailed analysis of HA – glycan interactions.

### Conformational dynamics of HA1 and HA2 subunits

To investigate the conformational changes in the HA protein of each strain, we also analyzed the RMSD of their HA1 and HA2 subunits (Table 1 and Figure S3). For the H1N1 strain (pdm09), overall RMSD fluctuations in the HA protein of pdm09 are primarily driven by conformational changes in the HA2 subunit. For the Feline/GA/2023 strain, both HA1 and HA2 contributed comparably to the observed structural fluctuation. For the Feline/YS/2023 strain, compared to HA2, the HA1 subunit exhibits greater conformational flexibility, contributing more significantly to the overall RMSD variation in the HA protein of Feline/YS/2023. For the Duck/YC/2022 strain, conformational changes in HA2 accounted for most of the observed structural fluctuations.

### Glycan conformational adaptation upon binding

To explore ligand conformational changes, we analyzed the ligand RMSD over the course of the simulation (Table 2 and Figure S4). Overall, α2,3-SLN exhibited more fluctuation than α2,6-SLN in the H1N1 (pdm09) complex. On the contrary, α2,6-SLN showed greater fluctuation than α2,3-SLN in the H5N1 (Feline/GA/2023, Feline/YS/2023 and Duck/YC/2022) complexes. Although the average ligand RMSD values were similar for the Feline/YS/2023 and Duck/YC/2022 complexes, the RMSD plots revealed that the α2,3-SLN maintained a more stable conformation, whereas the α2,6-SLN exhibited more fluctuations.

**Table 2.** Average RMSDs and angle θ (degrees) along with their standard deviations for the SLN ligand during MD simulations.

Virus – glycan complex	RMSD (Å)	Average θ
pdm09—α2,3-SLN	2.51 ± 0.62	158.86° ± 6.88°
pdm09—α2,6-SLN	1.24 ± 0.18	92.94° ± 5.38°
Feline/GA/2023—α2,3-SLN	1.81 ± 0.70	162.26° ± 5.88°
Feline/GA/2023—α2,6-SLN	3.28 ± 0.34	98.70° ± 6.07°
Feline/YS/2023—α2,3-SLN	2.72 ± 0.43	159.53° ± 6.24°
Feline/YS/2023—α2,6-SLN	2.44 ± 0.35	114.73° ± 13.71°
Duck/YC/2022—α2,3-SLN	2.83 ± 0.50	159.97° ± 5.68°
Duck/YC/2022—α2,6-SLN	2.41 ± 0.54	102.82° ± 13.34°

For a detailed analysis of conformational changes in the ligand, we measured the angle (θ) formed across its N-acetylneuraminic acid (NeuAc), Galactose (Gal) and N-acetylglucosamine (GalNAc) moieties (Figure S5). According to, the avian-type α2,3-SLN typically adopts a cone-like topology characterized by θ > 110°, while the human-type α2,6-SLN adopts an umbrella-like topology with θ < 110°. Consistent with previous studies [36–39], α2,3-SLN exhibited a cone-like topology in all complexes. Similarly, α2,6-SLN displayed the expected umbrella-like topology in all complexes except its complex with Feline/YS/2023 (Table 2 and Figure S6). In the case of Feline/YS/2023, high SD suggests substantial angular fluctuations, which may explain the slight deviation from the typical umbrella-like topology. The observed variations in angle θ correlated well with the RMSD values of the ligand, indicating a potential relationship between glycan conformation and structural stability.

### Binding free energy comparison of viral complexes with avian and human-type receptors

To characterize the binding affinities of each strain for avian (α2,3-SLN) and human (α2,6-SLN) glycans, we performed binding free energy calculations using the MM/PBSA method. As presented in Table 3, the binding free energies (ΔG) for both α2,3-SLN and α2,6-SLN complexes with each strain were calculated, along with their relative binding energy difference (ΔΔG). For comparative analyses across systems, the ΔΔG values were used. In the case of the H1N1 strain (pdm09), human-type α2,6-SLN complex showed higher binding affinity than avian-type α2,3-SLN complex with ΔΔG value of −2.74 kcal/mol, consistent with previous studies [18]. In contrast, for the H5N1 strains, avian-type α2,3-SLN complex displayed higher binding affinity than human-type α2,6-SLN complex. Specifically, ΔΔG values for Feline/GA/2023, Feline/YS/2023 and Duck/YC/2022 were found to be 3.20, 5.40 and 1.87 kcal/mol. Among H5N1 strains, the relative binding affinities decreased in the order of Feline/YS/2023 > Feline/GA/2023 > Duck/YC/2022.

**Table 3.** Binding free energies (ΔG, kcal/mol) and relative binding free energies (ΔΔG, kcal/mol) of the α2,3-SLN and α2,6-SLN complexes.

Strain	ΔG_α2,3-SLN_	ΔG_α2,6-SLN_	ΔΔG
pdm09	−38.15 ± 7.83	−40.89 ± 7.05	−2.74
Feline/GA/2023	−35.60 ± 8.15	−32.40 ± 6.28	+3.20
Feline/YS/2023	−40.40 ± 6.30	−35.00 ± 6.97	+5.40
Duck/YC/2022	−37.98 ± 6.41	−36.11 ± 6.04	+1.87

ΔΔG = ΔG_α2,6-SLN_ — ΔG_α2,3-SLN_.

### MM-PBSA based identification of residues associated with receptor binding differences

To explore the residues contributing to the difference in binding energy between α2,3-SLN and α2,6-SLN complexes, we performed a comparative analysis that included per-residue energy decomposition, binding mode from the final MD frame and hydrogen bond (H-bond) occupancy data.

**Figure 2.**
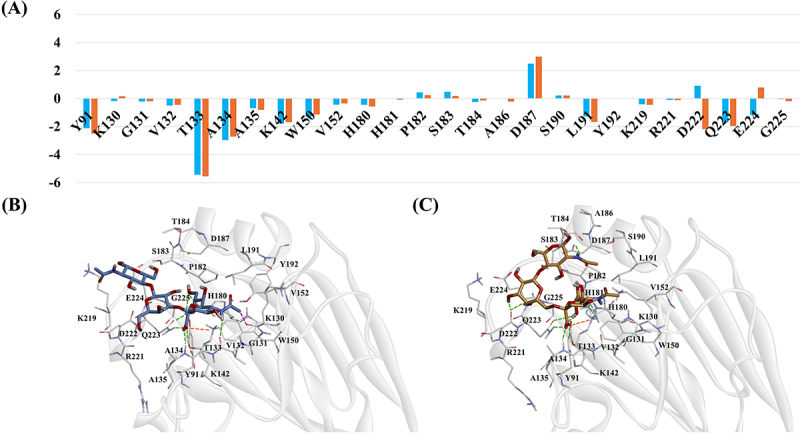
(A) Per-residue energy decomposition for pdm09 complexes based on MM/PBSA analysis. The X-axis represents individual residues and the Y-axis shows the energy contribution in kcal/mol. Orange bars correspond to the α2,6-SLN complex while blue bars represent the α2,3-SLN complex. (B–C) Binding modes of the (B) α2,3-SLN and (C) α2,6-SLN ligands from the final MD simulation frames. Ligands are shown as stick models and RBS residues are depicted as thick lines. Hydrogen bonds, pi-sigma interactions and electrostatic interactions are indicated by green, purple and orange dotted lines, respectively.

In the pdm09 strain, residues Y91, T133, A134, K142 and Q223 (H1 numbering) exhibited high energy contributions in both α2,3-SLN and α2,6-SLN complexes. These residues also formed H-bonds with occupancy values ≥50% during the simulation (Figure 2 and Table S2). Notably, significant differences were observed for residues D222 and E224 between the two complexes. D222 contributed favorably to binding in the α2,6-SLN complex but unfavorably in the α2,3-SLN complex. Conversely, E224 showed a favorable energy contribution in the α2,3-SLN complex but unfavorable in the α2,6-SLN complex. In the α2,6-SLN complex, D222 formed two high-occupancy H-bonds with the ligand. This is consistent with previous findings identifying D222 as a key residue for α2,6-SLN binding [40]. In contrast, in the α2,3-SLN complex, E224 formed two high-occupancy H-bonds with the ligand through its side chain.

**Figure 3.**
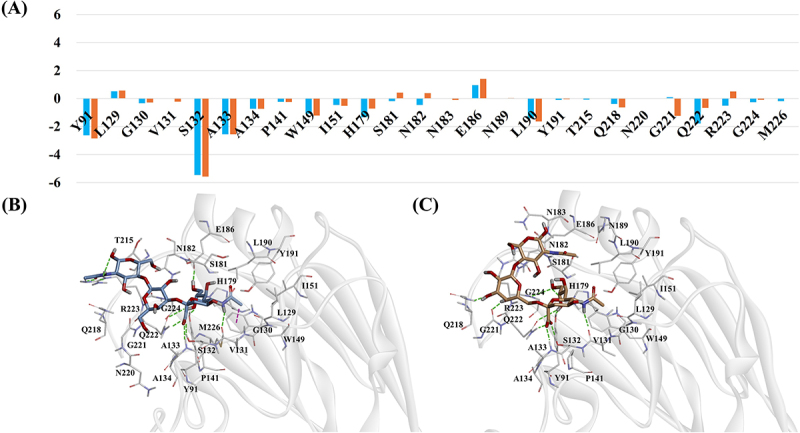
(A) Per-residue energy decomposition for Feline/GA/2023 complexes based on MM/PBSA analysis. The X-axis represents individual residues and the Y-axis shows the energy contribution in kcal/mol. Orange bars correspond to the α2,6-SLN complex while blue bars represent the α2,3-SLN complex. (B–C) Binding modes of the (B) α2,3-SLN and (C) α2,6-SLN ligands from the final MD simulation frames. Ligands are shown as stick models and RBS residues are depicted as thick lines. Hydrogen bonds and pi-sigma interactions are indicated by green and purple dotted lines, respectively.

In the Feline/GA/2023 strain, residues Y91, S132 and A133 (H5 numbering) showed significant energy contributions in both α2,3-SLN and α2,6-SLN complexes, each forming H-bonds with occupancy values exceeding 50% (Figure 3 and Table S3). Residue Q222 contributed more favorably to the binding energy in the α2,3-SLN complex than in the α2,6-SLN complex. In the α2,3-SLN complex, its side chain formed three H-bonds with the ligand. In contrast, in the α2,6-SLN complex, Q222 side chain formed only two H-bonds with the ligand. Residue G221 contributed more favorably in the α2,6-SLN complex. Its backbone formed an H-bond with the ligand showing an 82% occupancy. However, in the α2,3-SLN complex, this H-bond bond had a lower occupancy of 22%. Residue R223 exhibited a favorable energy contribution in the α2,3-SLN complex but was unfavorable in the α2,6-SLN complex. In the α2,3-SLN complex, its side chain formed a H-bond with 28% occupancy, whereas in the α2,6-SLN complex, the occupancy of this H-bond was below 10%. Residues S181 and N182 showed weak favorable contributions in the α2,3-SLN complex and unfavorable contributions in the α2,6-SLN complex. Consistently, H-bond analysis revealed very low occupancies (<10%) for both residues in both complexes.

**Figure 4.**
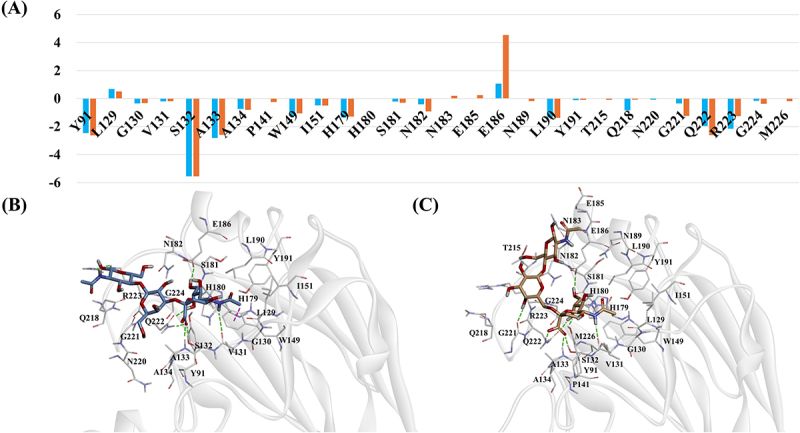
(A) Per-residue energy decomposition for Feline/YS/2023 complexes based on MM/PBSA analysis. The X-axis represents individual residues and the Y-axis shows the energy contribution in kcal/mol. Orange bars correspond to the α2,6-SLN complex while blue bars represent the α2,3-SLN complex. (B–C) Binding modes of the (B) α2,3-SLN and (C) α2,6-SLN ligands from the final MD simulation frames. Ligands are shown as stick models and RBS residues are depicted as thick lines. Hydrogen bonds and pi-sigma interactions are indicated by green and purple dotted lines, respectively.

In the case of the Feline/YS/2023 strain, residues Y91, S132 and A133 contributed significantly to the binding energy in both α2,3-SLN and α2,6-SLN complexes, with H-bond occupancies exceeding 69% (Figure 4 and Table S4). Residue Q218 showed a favorable contribution in the α2,3-SLN complex but negligible in the α2,6-SLN complex. In α2,3-SLN, its side chain formed one H-bond with the ligand (22% occupancy), whereas in α2,6-SLN, the occupancy of this H-bond was below 10%. Residue G221 contributed more favorably in the α2,6-SLN complex than in the α2,3-SLN complex, which correlates with a stronger H-bond interaction between its backbone and the ligand, reflected by H-bond occupancy of 76% in α2,6-SLN compared to 28% in α2,3-SLN. Q222 also contributed more favorably in the α2,6-SLN complex. Its side chain formed three H-bonds with the ligand, with higher occupancies, whereas in the α2,3-SLN complex, Q222 formed only two H-bonds with relatively lower occupancies. R223 contributed more favorably in the α2,3-SLN complex, establishing three H-bonds through both its backbone and side chain. In contrast, the α2,6-SLN complex exhibited only a single side-chain H-bond with lower occupancy.

**Figure 5.**
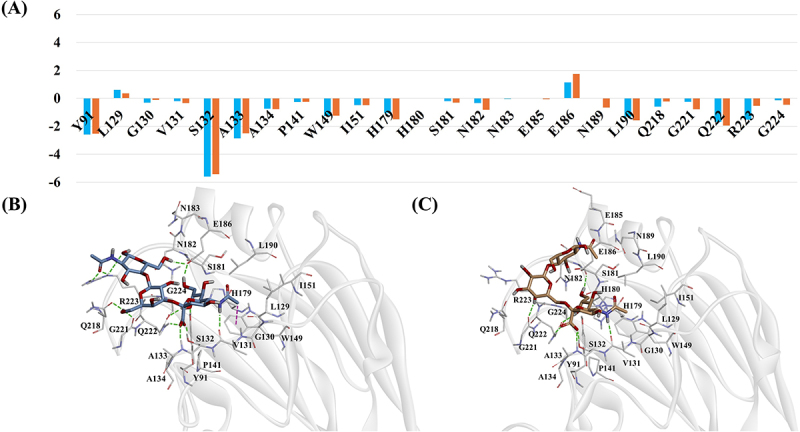
(A) Per-residue energy decomposition for Duck/YC/2022 complexes based on MM/PBSA analysis. The X-axis represents individual residues and the Y-axis shows the energy contribution in kcal/mol. Orange bars correspond to the α2,6-SLN complex while blue bars represent the α2,3-SLN complex. (B–C) Binding modes of the (B) α2,3-SLN and (C) α2,6-SLN ligands from the final MD simulation frames. Ligands are shown as stick models and RBS residues are depicted as thick lines. Hydrogen bonds and pi-sigma interactions are indicated by green and purple dotted lines, respectively.

In the case of the Duck/YC/2022 strain, residues Y91, S132 and A133 contributed significantly to the binding energy in both α2,3-SLN and α2,6-SLN complexes, each forming H-bonds with occupancies exceeding 50% (Figure 5 and Table S5). Residue Q222 showed a slightly more favorable contribution in the α2,6-SLN complex compared to the α2,3-SLN complex. In α2,6-SLN, its side chain formed two H-bonds with the ligand, with occupancies of 78% and 48%. In contrast, in α2,3-SLN, this residue formed two H-bonds with slightly lower occupancies of 60% and 37%. R223 exhibited a more favorable energy contribution in the α 2,3-SLN complex, establishing three H-bonds through its backbone and side chain with high occupancy. In contrast, the α2,6-SLN complex showed only weak interactions with occupancies below 10%.

### Structural and energetic comparison of HA–α2,3-SLN complexes

We investigated the differences among three H5N1 strains (Feline/GA/2023, Feline/YS/2023 and Duck/YC/2022) which demonstrated relatively higher binding affinities with α2,3-SLN compared to α2,6-SLN in the MM/PBSA calculations. Superposition of the final MD structures revealed overall structural similarity, but notable differences in the composition and conformation of the RBS residues and the ligand conformations.

Across all strains, the RBS consistently included residues Y91, L129, G130, V131, S132, A133, A134, W149, I151, H179, S181, N182, E186, L190, Q218, G221, Q222, R223 and G224. In addition to these conserved residues, strain-specific RBS differences were identified. T215 and M226 were unique to Feline/GA/2023, H180 was present only in Feline/YS/2023, and N183 was unique to Duck/YC/2022. Residue P141 was common to Feline/GA/2023 and Duck/YC/2022, while Y191 and N220 were common to Feline/GA/2023 and Feline/YS/2023 (Figures 3–5).

To assess the conformational flexibility of the RBS, we calculated the RMSF values for each strain. Overall, the RBS flexibility decreased in the order of Feline/GA/2023 > Duck/YC/2022 > Feline/YS/2023 (Figure S7). This trend in flexibility correlated with the MM/PBSA binding energies of their complexes, Feline/GA/2023 (−35.60 kcal/mol), Duck/YC/2022 (−37.98 kcal/mol) and Feline/YS/2023 (−40.40 kcal/mol), suggesting that reduced flexibility enhances binding (Table 3).

Conformational variations were also observed in the α2,3-SLN ligand (Figure S8). While the Neu5Ac moiety remained stable, the Gal and GalNAc moieties exhibited varying degrees of extension, with angle θ correlating with RBS flexibility and binding energy (Table 2).

In addition to examining conformational changes within the RBS residues and the ligand, we further explored their impact on protein-ligand interactions. In the final MD structures, residues V131, S132, A133, E186, Q222 and R223 were common interaction partners for all three strains, Feline/GA/2023 formed additional H-bonds with H179 and Y91, and Duck/YC/2022 with Q218 and Y91 (Figures 3–5). These findings indicate that strain-specific conformational variations in both the protein and ligand primarily account for the observed differences in α2,3-SLN binding.

## Discussion

Influenza A viruses continue to pose a major threat to both animal and human health due to their capacity for cross-species transmission and rapid genetic evolution. Among these, HPAI H5N1 viruses are of particular concern due to their increasing zoonotic potential and high fatality rates. Understanding the molecular determinants that govern host specificity and transmissibility is therefore critical for assessing pandemic risk and guiding surveillance strategies.

Among these, HA – SA interactions at the entry stage represent a key determinant of viral transmissibility. Avian influenza viruses preferentially bind to α2,3-linked SA receptors. By contrast, human influenza viruses such as H1, H2, and H3 subtypes preferentially bind to α2,6-linked SA receptors [8]. This receptor binding specificity largely explains why most HPAI H5N1 viruses demonstrate strong affinity for avian-type α2,3-linked receptors and limited potential for efficient human-to-human spread [7,8].

Molecular studies have identified key HA mutations, including S123P, S133A, T156A, Q222L and G224S (H5 numbering) that can shift receptor preference toward human-type α2,6 receptors and thereby enhance cross-species transmission [9–17]. In a previous study by Kim et al. [6], we characterized the first HPAI H5N1 strains associated with fatal cluster infections in felines in the Republic of Korea. Notably, the Korean strains carried the S123P, S133A, and T156A mutations but lacked the critical Q222L and G224S mutations, indicating that despite their pathogenicity in mammals, they were unlikely to have acquired human-type receptor affinity based solely on genetic evidence. However, a key limitation of that study was the absence of direct experimental validation of receptor-binding properties.

To overcome this, the present study combined experimental receptor binding assays with computational analyses to directly evaluate the receptor binding profiles. Using solid-phase binding assays, we examined three H5N1 isolates (two feline-derived and one avian) alongside a human H1N1 reference strain, testing their affinity for both avian-type (α2,3-SLN) and human-type (α2,6-SLN) receptors. Consistent with previous findings, the H1N1 (pdm09) strain bound strongly to α2,6-linked SA receptors. In contrast, all three H5N1 strains (Feline/GA/2023, Feline/YS/2023 and Duck/YC/2022) exhibited strong binding to α2,3-linked SA receptors, while showing no detectable binding to α2,6-linked SA receptors. These results provide experimental evidence that feline-derived H5N1 isolates retain avian-type receptor specificity.

The structural modeling, molecular dynamics simulations, and MM-PBSA per-residue energy decomposition performed in this study provide computational insight into receptor binding interactions and energetics; however, these approaches are inherently predictive and do not establish structure – function relationships. Accordingly, the identified structural features and residues should be regarded as potential contributors whose functional relevance will require validation through experimental studies. MD simulations and MM/PBSA free energy calculations revealed that the H1N1–α2,6 complex was structurally more stable and energetically more favorable than H1N1–α2,3, consistent with its human-type receptor preference. Conversely, all H5N1 strains formed more stable and energetically more favorable complexes with α2,3-SLN than with α2,6-SLN, aligning with their expected avian-type receptor preference. Per-residue energy decomposition and H-bond occupancy analyses further revealed key RBS residues underlying the differential binding. Across all three H5N1 strains, Y91, S132 and A133 consistently contributed to receptor engagement. Additional strain-specific effects were observed for Q218, G221, Q222, and R223, which differentially influenced receptor preference. Notably, Q222 emerged as a critical determinant for α2,6 binding. Although A133 has been reported to facilitate α2,6 binding, our results showed no significant difference in its contribution between α2,3 and α2,6 interactions, aligning with prior genetic analyses [6]. Thus, Y91 and S132 serve as common contributors, while Q218, G221, Q222 and R223 act as strain-specific modulators of binding affinity and host receptor specificity.

We also assessed the conformational dynamics of both HA and the glycans. RMSD analysis showed strain-dependent flexibility in the HA1 and HA2 subunits, while glycan dynamics revealed distinct behaviors between avian and human receptors. In the H1N1 complex, α2,3-SLN exhibited greater fluctuation than α2,6-SLN, whereas in the H5N1 complexes, α2,6-SLN was more dynamic than α2,3-SLN. In agreement with previous studies, α2,3-SLN consistently adopted a cone-like topology (θ > 110°), whereas α2,6-SLN displayed an umbrella-like topology (θ < 110°) [36–39]. Overall, these topologies remained consistent across complexes but showed strain-dependent variations in θ values. Structural superposition of final MD complexes with α2,3-SLN further demonstrated subtle differences in RBS composition and glycan positioning, which influenced binding energetics across H5N1 strains. Reduced flexibility within the RBS correlated with stronger binding, emphasizing the significance of local structural rigidity for effective receptor recognition. Overall, our results highlight the intricate interplay between protein flexibility, ligand topology and intermolecular interactions in determining receptor binding strength and specificity. These findings elucidate how subtle conformational differences at the molecular interface drive HA-receptor interaction and viral host preference.

Collectively, our structural and energetic analyses reveal that while H5N1 viruses are known to preferentially bind avian-type α2,3-linked sialic acid receptors, the increasing occurrence of mammalian infections underscores the need for host- and context-specific reassessment of receptor binding properties. In this study, we compared recent feline-derived clade 2.3.4.4b H5N1 viruses from South Korea with a genetically related avian strain to evaluate whether cross-species transmission was accompanied by altered receptor specificity. The observed retention of avian-type receptor preference suggests that the jump from birds to felines occurred without a substantial shift toward human-type α2,3 receptor binding.

These results are consistent with assessments by WHO and other experts that the absence of adaptive receptor binding changes continues to limit zoonotic potential [41]. However, the increasing number of H5N1 infections in mammals worldwide has raised legitimate concerns about viral adaptation. While our study strengthens the evidence for avian receptor preference, its limitations include a narrow focus on HA – SA binding, lack of *in vivo* transmissibility data and reliance on monomer-based computational analyses. Despite these limitations, receptor binding assays coupled with computational modeling provide valuable insights into cross-species transmission potential. Our ongoing surveillance of Korean HPAI strains aims to identify early indicators of increased human risk. These efforts are critical for pandemic preparedness and supporting rapid public health responses to emerging zoonotic threats.

In conclusion, despite high virulence in felines, the tested H5N1 strains exhibit strict avian receptor specificity, suggesting a limited risk of sustained human transmission at present. Continued genomic and functional surveillance is essential to promptly detect potential receptor binding shifts that could signal emerging pandemic threats.

## Supplementary Material

Supplementary data 2 _cleancopy.docx

Supplementary data 1_cleancopy.docx

## Data Availability

The data presented in this study are available in the supplementary materials.
