# Supplementary material for: Analyses of receptor binding specificity of highly pathogenic avian influenza A (H5N1) viruses isolated from felines in South Korea, 2023
Source: Virulence. 2026 Feb 23;17(1):2636350. doi: 10.1080/21505594.2026.2636350 (PMC12940103; doi:10.1080/21505594.2026.2636350)
Supplement: Supplementary data 2 _cleancopy.docx [file KVIR_A_2636350_SM3295.docx]

**Supplementary data 2**

**HA protein sequences and GISAID details for all influenza viruses**

>A/feline/Korea/M305-7/2023|H5N1|HA|EPI_ISL_18819807

MENIVLLLATISLVKSDQICIGYHANNSTEQVDTIMEKNVTVTHAQDILEKTHNGKLCDLNGVKPLILKDCSVAGWLLGNPMCDEFIRVPEWSYIVERNNPANDLCYPGSLNDYEELKHLLSRINHFEKILIIPKSSWPNHETSLGVSAACPYQGAPSFFRNVVWLIKKDDAYPTIKISYNNTNREDLLILWGIHHSNNAEEQTNLYKNPTTYISVGTSTLNQRLVPKIATRSQVNGQRGRMDFFWTILKPDDAIHFESNGNFIAPEYAYKIVKKGDSTIMKSGVEYGHCNTKCQTPVGAINSSMPFHNIHPLTIGECPKYVKSNKLVLATGLRNSPLREKRRKRGLFGAIAGFIEGGWQGMVDGWYGYHHSNEQGSGYAADKESTQKAIDGVTNKVNSIIDKMNTQFEAVGREFNNLERRIENLNKKMEDGFLDVWTYNAELLVLMENERTLDFHDSNVKNLYDKVRLQLRDNAKELGNGCFEFYHKCDNECMESVRNGTYYYPQYSEEARLKREEISGVKLESIGTYQILSIYSTAASSLALAIMMAGLSLWMCSNGSLQCRICI

>A/feline/Korea/M302-6/2023|H5N1|HA|EPI_ISL_18819809

MENIVLLLATISLVKSDQICIGYHANNSTEQVDTIMEKNVTVTHAQDILEKTHNGKLCDLNGVKPLILKDCSVAGWLLGNPMCDEFIRVPEWSYIVERDNPANDLCYPGSLNDYEELKHLLSRINHFEKILIIPKSSWPNHETSLGVSAACPYQGAPSFFRNVVWLIKKDDAYPTIKISYNNTNREDLLILWGIHHSNNAEEQTNLYKNPTTYISVGTSTLNQRLVPKIATRSQVNGQRGRMDFFWTILKPDDAIHFESNGNFIAPEYAYKIVKKGDSTIMKSGVEYGHCNTKCQTPVGAINSSMPFHNIHPLTIGECPKYVKSNKLVLATGLRNSPLREKRRKRGLFGAIAGFIEGGWQGMVDGWYGYHHSNEQGSGYAADKESTQKAIDGVTNKVNSIIDKMNTQFEAVGREFNNLERRIENLNKKMEDGFLDVWTYNAELLVLMENERTLDFHDSNVKNLYDKVRLQLRDNAKELGNGCFEFYHKCDNECMESVRNGTYYYPQYSEEARLKREEISGVKLESIGTYQILSIYSTAASSLALAIMMAGLSLWMCSNGSLQCRICI

>A/duck/Korea/H493/2022|H5N1|HA|EPI_ISL_15647834

MENIVLLLAIVNLVKSDQICIGYHANNSTEQVDTIMEKNVTVTHAQDILEKTHNGKLCDLNGVKPLILKDCSVAGWLLGNPMCDEFIRVPEWSYIVERANPANDLCYPGSLNDYEELKHLLSRINHFEKILIIPKSSWPNHETSLGVSAACPYQGAPSFFRNVVWLIKKDDAYPTIKISYNNTNREDLLILWGIHHSNNAEEQTNLYKNPTTYISVGTSTLNQRLVPKIATRSQVNGQRGRMDFFWTILKPDDAIHFESNGNFIAPEYAYKIVKKGDSTIMKSGVEYGHCNTKCQTPVGAINSSMPFHNIHPLTIGECPKYVKSNKLVLATGLRNSPLREKRRKRGLFGAIAGFIEGGWQGMVDGWYGYHHSNEQGSGYAADKESTQKAIDGVTNKVNSIIDKMNTQFEAVGREFNNLERRIENLNKKMEDGFLDVWTYNAELLVLMENERTLDFHDSNVKNLYDKVRLQLRDNAKELGNGCFEFYHKCDNECMESVRNGTYYYPQYSEEARLKREEISGVKLESIGTYQILSIYSTAASSLALAIMMAGLSLWMCSNGSLQCRICI

>A/California/04/2009|H5N1|HA|EPI_ISL_29573

MKAILVVLLYTFATANADTLCIGYHANNSTDTVDTVLEKNVTVTHSVNLLEDKHNGKLCKLRGVAPLHLGKCNIAGWILGNPECESLSTASSWSYIVETPSSDNGTCYPGDFIDYEELREQLSSVSSFERFEIFPKTSSWPNHDSNKGVTAACPHAGAKSFYKNLIWLVKKGNSYPKLSKSYINDKGKEVLVLWGIHHPSTSADQQSLYQNADTYVFVGSSRYSKKFKPEIAIRPKVRDQEGRMNYYWTLVEPGDKITFEATGNLVVPRYAFAMERNAGSGIIISDTPVHDCNTTCQTPKGAINTSLPFQNIHPITIGKCPKYVKSTKLRLATGLRNIPSIQSRGLFGAIAGFIEGGWTGMVDGWYGYHHQNEQGSGYAADLKSTQNAIDEITNKVNSVIEKMNTQFTAVGKEFNHLEKRIENLNKKVDDGFLDIWTYNAELLVLLENERTLDYHDSNVKNLYEKVRSQLKNNAKEIGNGCFEFYHKCDNTCMESVKNGTYDYPKYSEEAKLNREEIDGVKLESTRIYQILAIYSTVASSLVLVVSLGAISFWMCSNGSLQCRICI
