# Supplementary material for: Analyses of receptor binding specificity of highly pathogenic avian influenza A (H5N1) viruses isolated from felines in South Korea, 2023
Source: Virulence. 2026 Feb 23;17(1):2636350. doi: 10.1080/21505594.2026.2636350 (PMC12940103; doi:10.1080/21505594.2026.2636350)
Supplement: Supplementary data 1_cleancopy.docx [file KVIR_A_2636350_SM3294.docx]

**Supplementary data 1**

**Table S1.** Overview of influenza virus strains used in this study.

| Virus Strain | Host origin | Geographical location | Collection  year | Subtype | Genetic clade | GISAID accession No. | Abbreviation |
| --- | --- | --- | --- | --- | --- | --- | --- |
| A/feline/Korea/M305-7/2023 | Feline | South Korea | 2023 | H5N1 | 2.3.4.4b | EPI_ISL_18819807 | Feline/GA/2023 |
| A/feline/Korea/M302-6/2023 | Feline | South Korea | 2023 | H5N1 | 2.3.4.4b | EPI_ISL_18819809 | Feline/YS/2023 |
| A/duck/Korea/H493/2022 | Duck | South Korea | 2022 | H5N1 | 2.3.4.4b | EPI_ISL_15647834 | Duck/YC/2022 |
| A/California/04/2009 | Human | USA | 2009 | H1N1 | 6B.1 | EPI_ISL_29573 | pdm09 |

**Table S2.** Residues exhibiting H-bond occupancies greater than 10% with ligands in pdm09 complexes.

| pdm09─α2,3-SLN | | pdm09─α2,6-SLN | |
| --- | --- | --- | --- |
| Residue | Occupancy (%) | Residue | Occupancy (%) |
| ALA134-Main-N | 91 | THR133-Side-OG1 | 91 |
| THR133-Side-OG1 | 89 | TYR91-Side-OH | 89 |
| LYS142-Side-NZ | 78 | ALA134-Main-N | 89 |
| TYR91-Side-OH | 74 | ASP222-Main-O | 87 |
| GLU224-Side-OE1 | 68 | GLN223-Side-NE2 | 73 |
| GLN223-Side-OE1 | 50 | ASP222-Side-OD | 70 |
| GLN223-Side-NE2 | 50 | LYS142-Side-NZ | 57 |
| GLU224-Side-OE2 | 46 | ASP187-Side-OD | 51 |
| VAL132-Main-O | 38 | VAL132-Main-O | 48 |
| LYS219-Side-NZ | 25 | GLN223-Side-OE1 | 36 |
| ASP222-Main-O | 19 | LYS219-Side-NZ | 29 |
| LYS130-Main- O | 17 |  |  |

**Table S3.** Residues exhibiting H-bond occupancies greater than 10% with ligands in Feline/GA/2023 complexes.

| Feline/GA/2023─α2,3-SLN | | Feline/GA/2023─α2,6-SLN | |
| --- | --- | --- | --- |
| Residue | Occupancy (%) | Residue | Occupancy (%) |
| TYR91-Side-OH | 96 | TYR91-Side-OH | 96 |
| SER132-Side-OG | 94 | SER132-Side-OG | 93 |
| GLU186-Side-OE2 | 93 | ALA133-Main-N | 87 |
| ALA133-Main-N | 82 | GLY221-Main-O | 82 |
| GLN222-Side-NE2 | 62 | GLN222-Side-NE2 | 79 |
| VAL131-Main-O | 57 | VAL131-Main-O | 76 |
| GLN222-Side-OE1 | 32 | GLU186-Side-OE | 56 |
| ARG223-Side-NH2 | 28 | GLN218-Side- OE1 | 31 |
| GLY221-Main-O | 22 | GLN222-Side-OE1 | 12 |
| GLN222-Side-CD | 22 |  |  |
| GLU186-Side-OE1 | 19 |  |  |
| GLN218-Side-NE2 | 13 |  |  |

**Table S4.** Residues exhibiting H-bond occupancies greater than 10% with ligands in Feline/YS/2023 complexes.

| Feline/YS/2023─α2,3-SLN | | Feline/YS/2023─α2,6-SLN | |
| --- | --- | --- | --- |
| Residue | Occupancy (%) | Residue | Occupancy (%) |
| TYR91-Side-OH | 95 | TYR91-Side-OH | 97 |
| SER132-Side-OG | 95 | SER132-Side-OG | 89 |
| ALA133-Main-N | 91 | GLU186-Side-OE2 | 85 |
| GLU186-Side-OE1 | 86 | GLN222-Side-NE2 | 84 |
| GLN222-Side-NE2 | 65 | GLY221-Main-O | 76 |
| VAL131-Main-O | 60 | ALA133-Main-N | 69 |
| ARG223-Side-NH2 | 45 | VAL131-Main-O | 67 |
| GLN222-Side-OE1 | 40 | GLN222-Side-OE1 | 65 |
| ARG223-Main-N | 30 | ARG223-Side-NH1 | 28 |
| GLY221-Main-O | 28 | GLN222-Side-CD | 11 |
| ARG223-Side-NE | 27 |  |  |
| GLN218-Side-NE2 | 22 |  |  |

**Table S5.** Residues exhibiting H-bond occupancies greater than 10% with ligands in Duck/YC/2022 complexes.

| Duck/YC/2022─α2,3-SLN | | Duck/YC/2022─α2,6-SLN | |
| --- | --- | --- | --- |
| Residue | Occupancy (%) | Residue | Occupancy (%) |
| TYR91-Side-OH | 95 | TYR91-Side-OH | 96 |
| SER132-Side-OG | 95 | SER132-Side-OG | 91 |
| ALA133-Main-N | 92 | GLU186-Side-OE2 | 79 |
| GLU186-Side-OE2 | 84 | GLN222-Side-NE2 | 78 |
| VAL131-Main-O | 63 | ALA133-Main-N | 74 |
| GLN222-Side-NE2 | 60 | VAL131-Main-O | 66 |
| ARG223-Side-NH2 | 40 | GLY221-Main-O | 62 |
| GLN222-Side-OE1 | 37 | GLN222-Side-OE1 | 48 |
| GLY221-Main-O | 35 | ASN183-Side-ND2 | 17 |
| ARG223-Main-N | 22 | GLN218-Side-OE1 | 15 |
| GLN218-Side-NE2 | 21 |  |  |
| GLU216-Side-OE1 | 18 |  |  |
| ARG223-Side-NE | 15 |  |  |


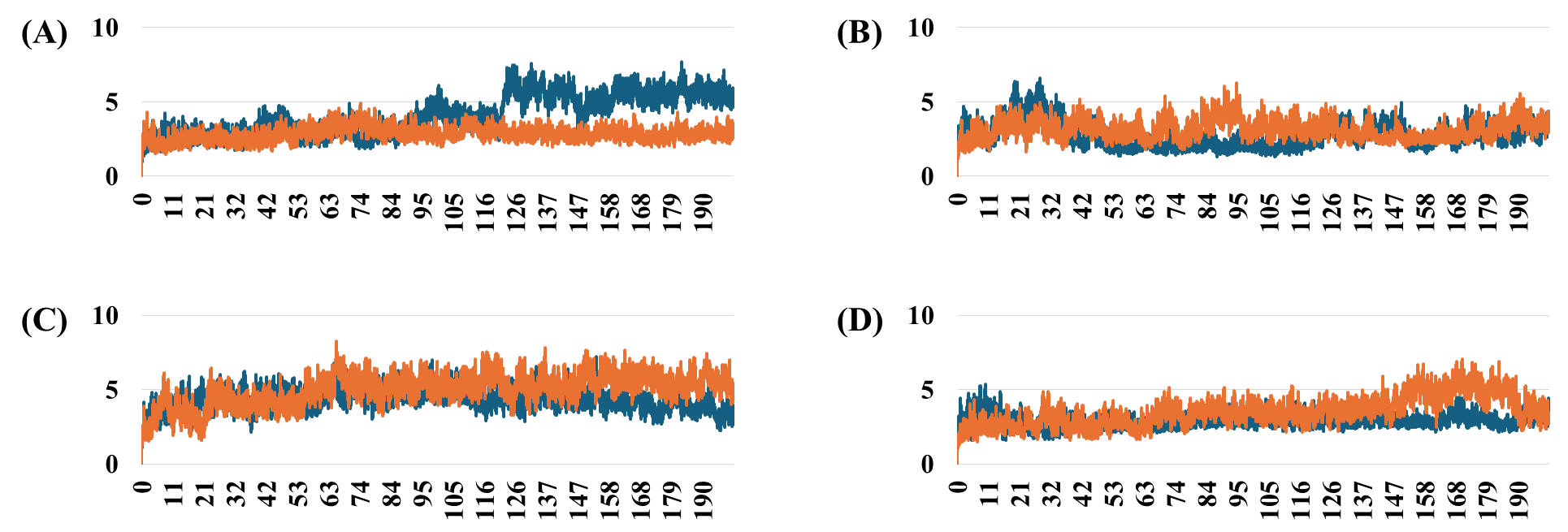


**Figure S1.** Time evolution of RMSD of the HA protein backbone atoms. (A) pdm09, (B) Feline/GA/2023 (C) Feline/YS/2023 and (D) Duck/YC/2022. The X axis indicates the time (ns) and Y axis indicates the RMSD (Å). Orange line represents the α2,6-SLN complex and blue line represents the α2,3-SLN complex.

**
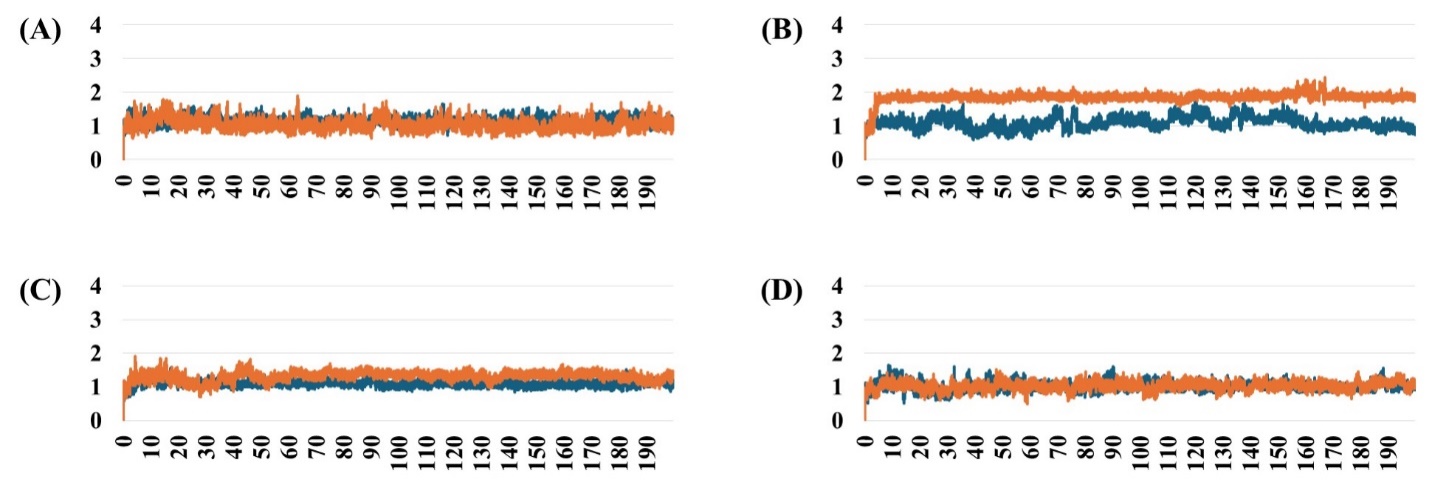
**

**Figure S2.** Time evolution of RMSD of the RBS residues. (A) pdm09, (B) Feline/GA/2023 (C) Feline/YS/2023 and (D) Duck/YC/2022. The X axis indicates the time (ns) and Y axis indicates the RMSD (Å). Orange line represents the α2,6-SLN complex and blue line represents the α2,3-SLN complex.


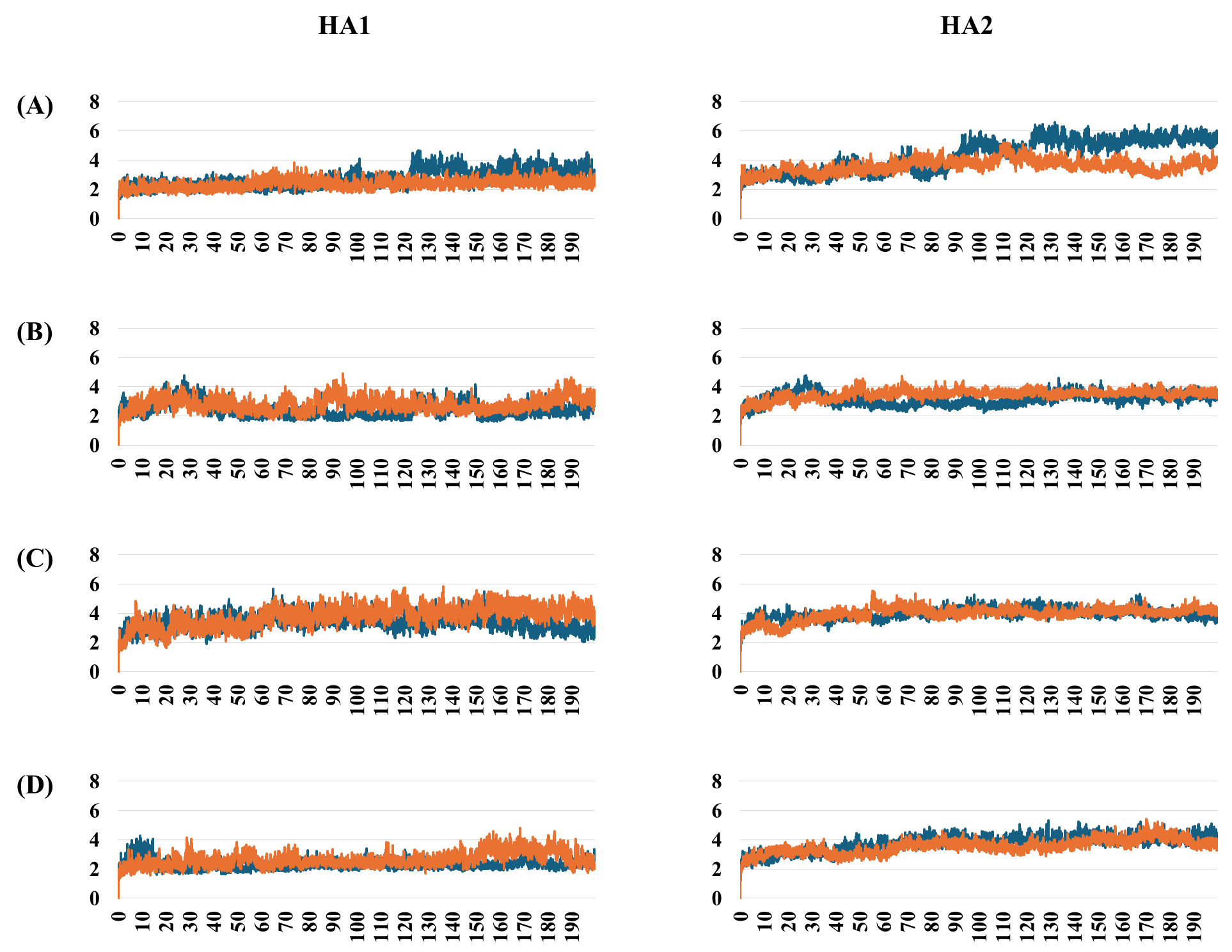


**Figure S3.** Time evolution of RMSD of the HA1 and HA2 subunits. (A) pdm09, (B) Feline/GA/2023 (C) Feline/YS/2023 and (D) Duck/YC/2022. The X axis indicates the time (ns) and Y axis indicates the RMSD (Å). Orange line represents the α2,6-SLN complex and blue line represents the α2,3-SLN complex.

**
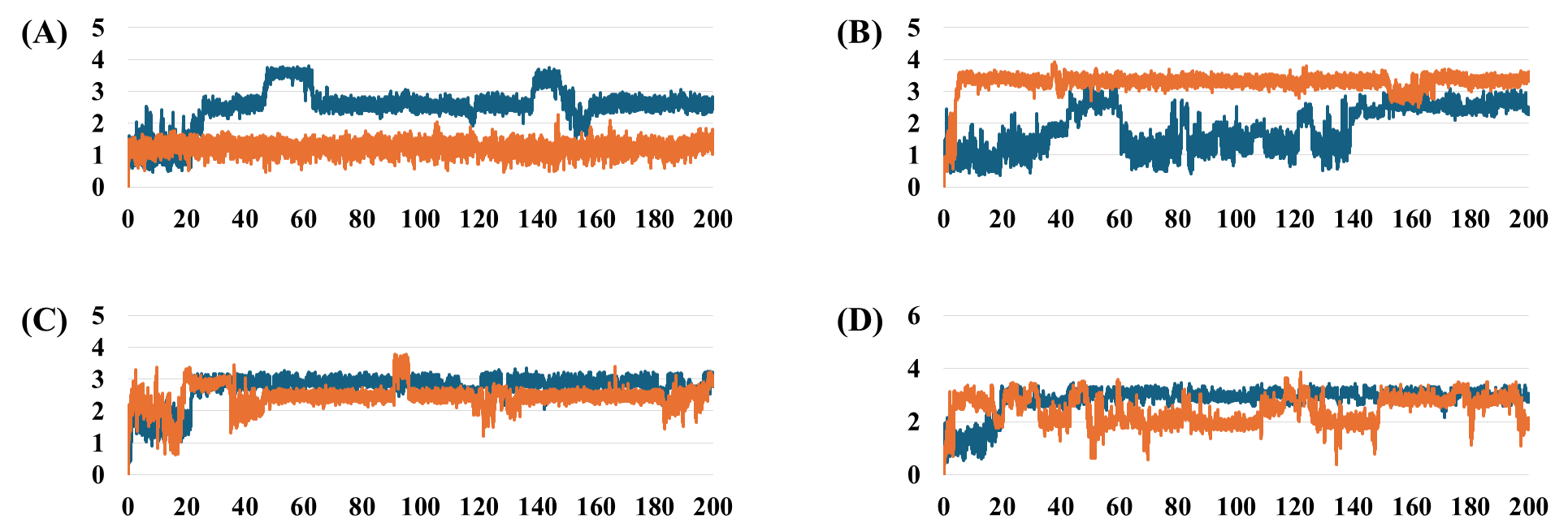
**

**Figure S4.** Time evolution of RMSD of the ligand. (A) pdm09, (B) Feline/GA/2023 (C) Feline/YS/2023 and (D) Duck/YC/2022. The X axis indicates the time (ns) and Y axis indicates the RMSD (Å). Orange line represents the α2,6-SLN complex and blue line represents the α2,3-SLN complex.


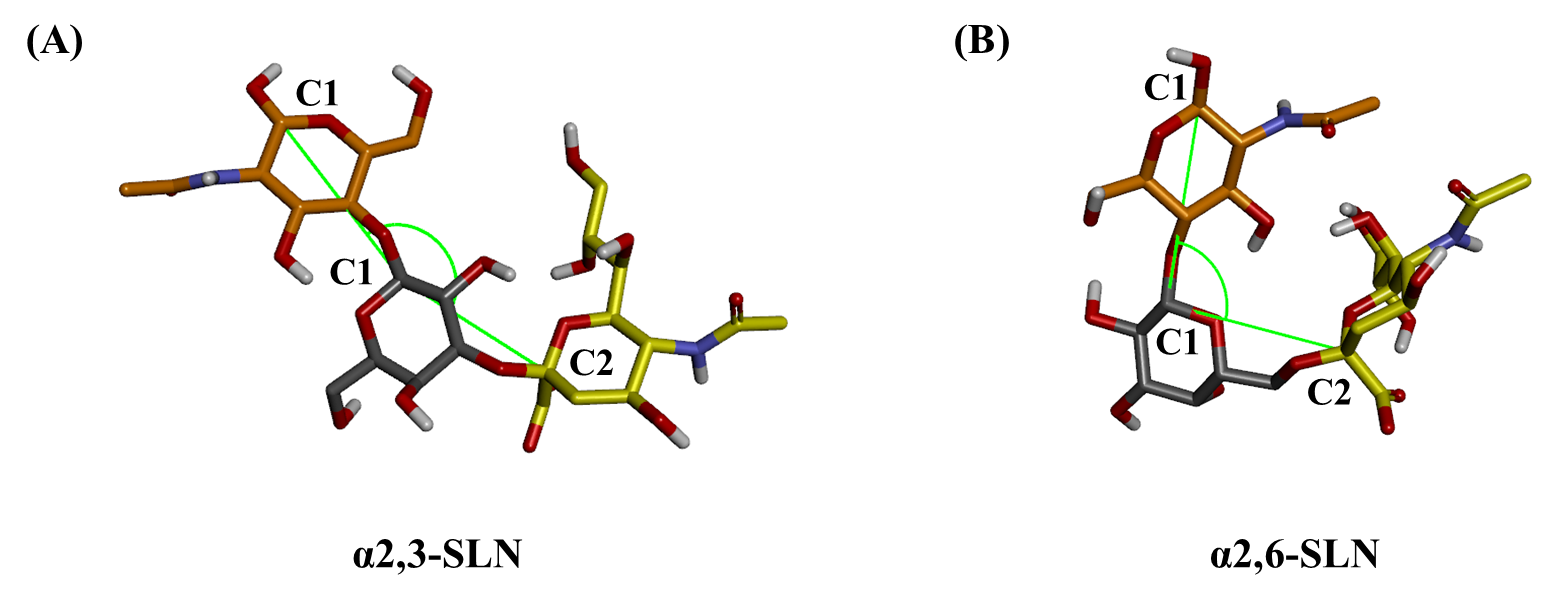


**Figure S5.** Illustration of angle θ in (A) α2,3-SLN and (B) α2,6-SLN. Angle θ defined by the atoms C2 of NeuAc, C1 of Gal and GalNAc moieties reflects the overall topology of the glycan.

**
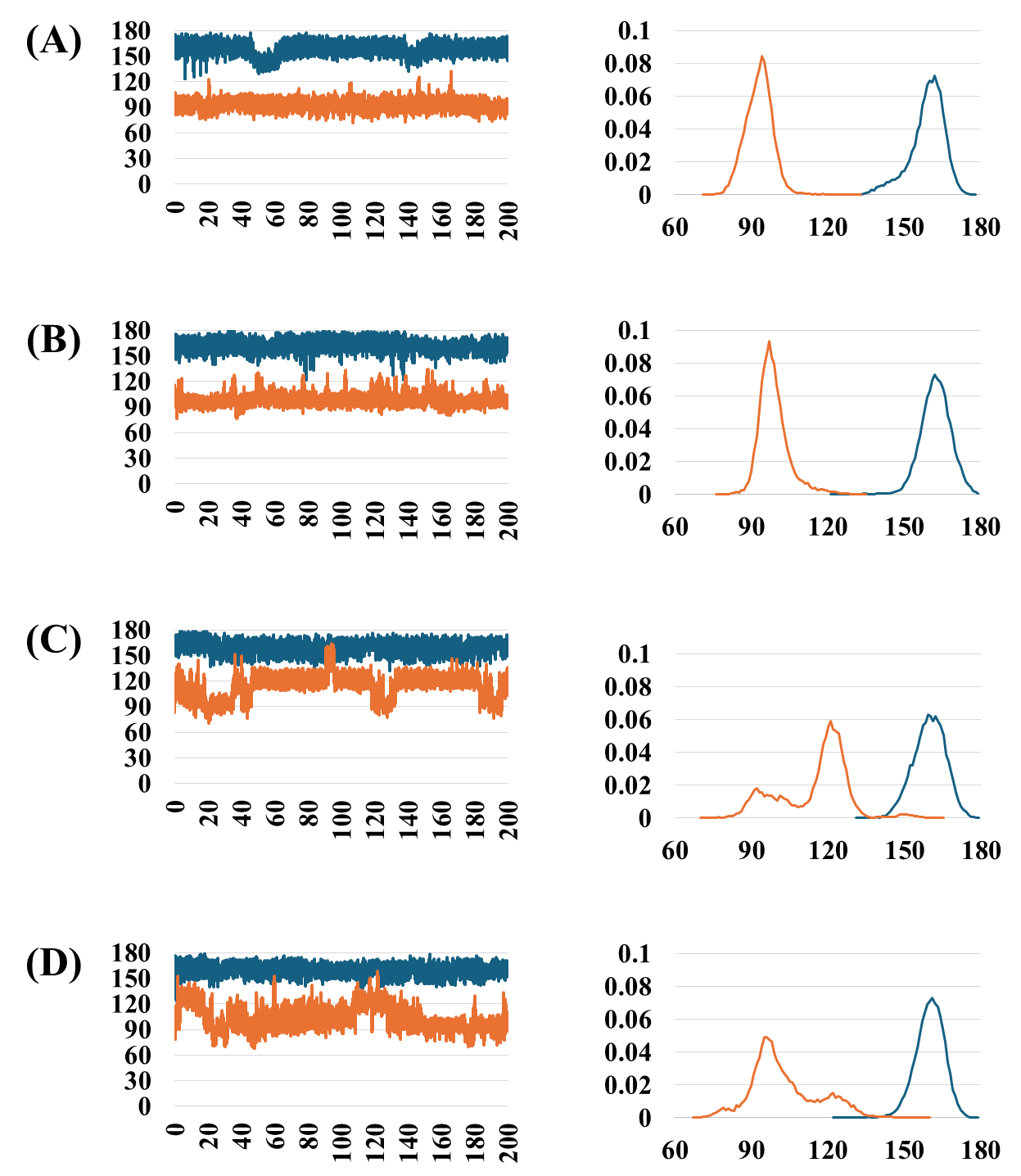
**

**Figure S6.** Time evolution (left panel) and probability distribution (right panel) of the angle θ in the ligand. (A) pdm09, (B) Feline/GA/2023 (C) Feline/YS/2023 and (D) Duck/YC/2022. In the left panel, the X-axis represents simulation time (ns) and the Y-axis shows angle θ (degrees). In the right panel, the X-axis indicates angle θ (degrees) and the Y-axis shows the probability distribution. In both panels, the orange line corresponds to the α2,6-SLN complex, and the blue line represents the α2,3-SLN complex.


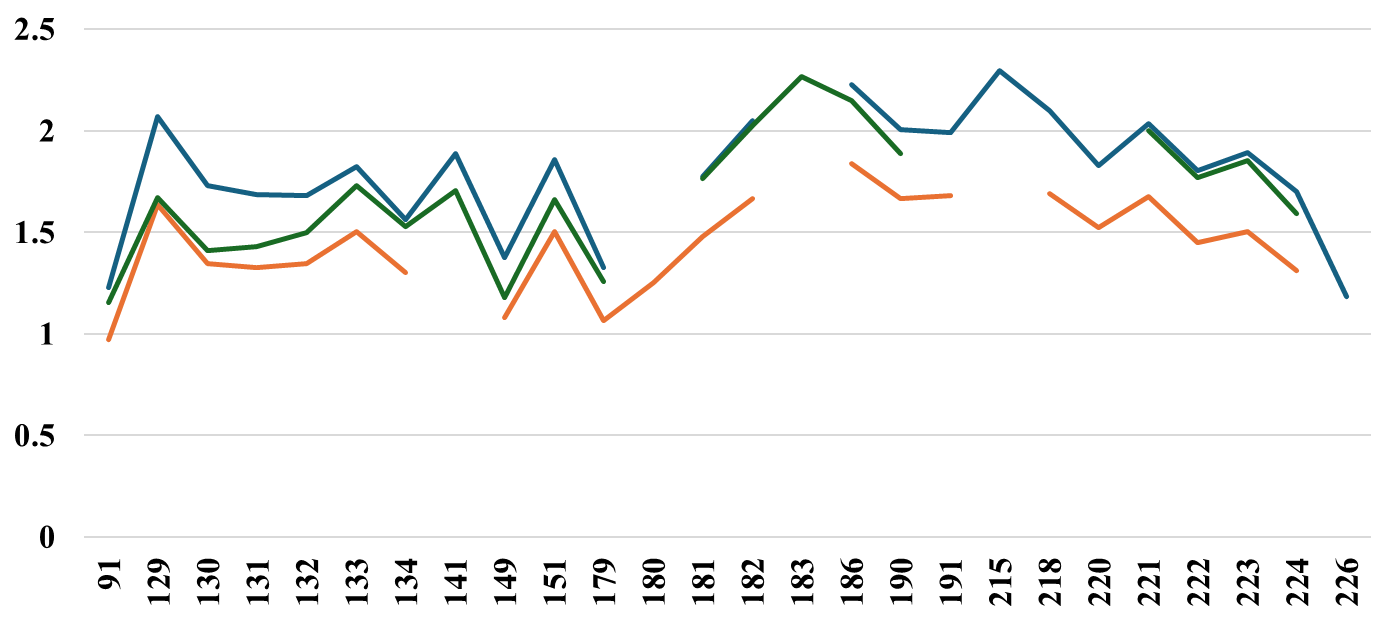


**Figure S7.** RMSF of RBS residues in α2,3-SLN complexes of three H5N1 strains. The X-axis denotes the individual residues and the Y-axis shows the RMSF values (Å). Feline/GA/2023, Feline/YS/2023 and Duck/YC/2022 strains are represented by blue, orange and green lines, respectively.

**
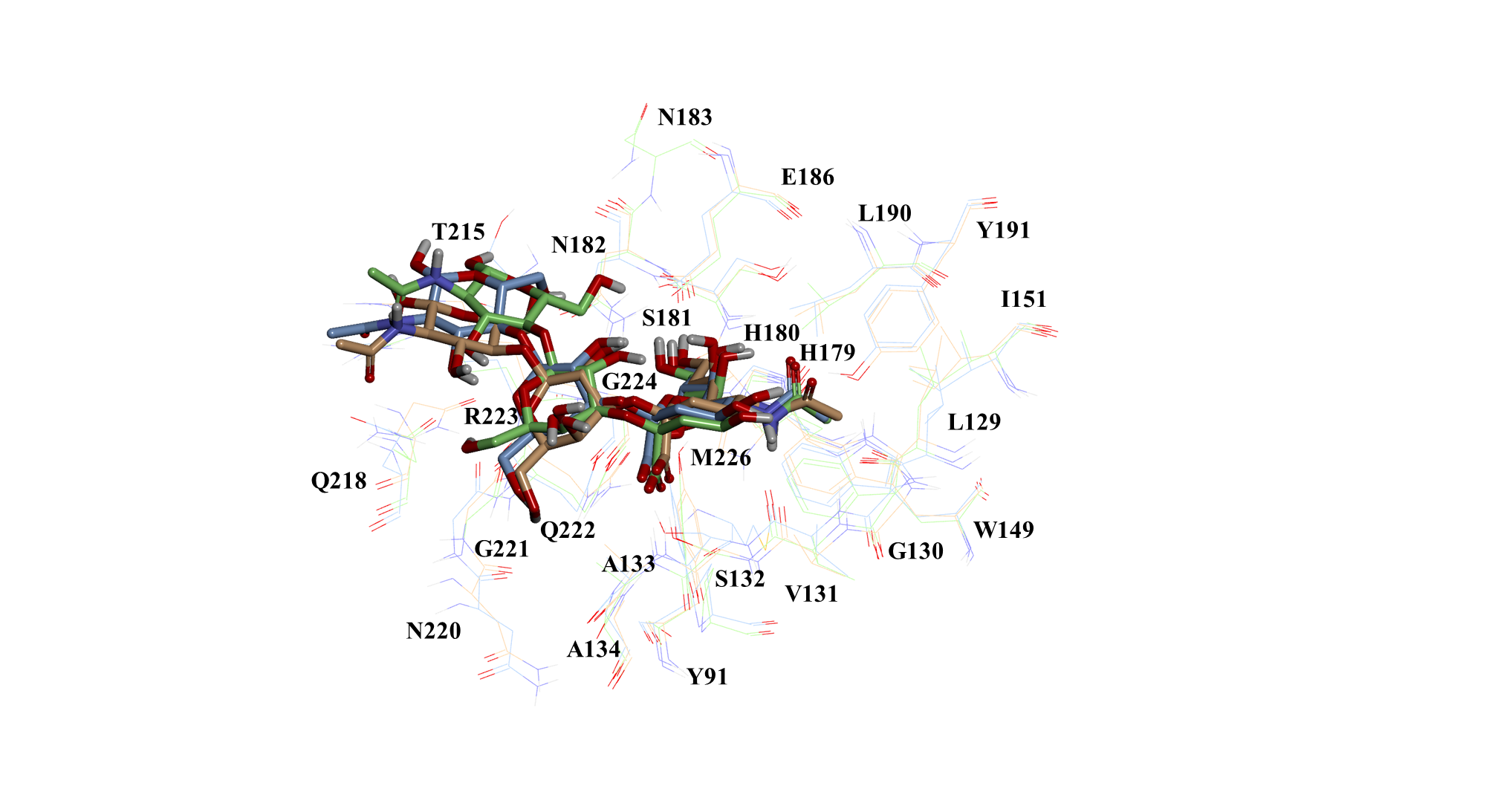
**

**Figure S8.** Conformational variations of the α2,3-SLN ligand in complexes with different H5N1 strains. Feline/GA/2023, Feline/YS/2023 and Duck/YC/2022 strains are represented by blue, orange and green lines, respectively.
